# The Late Dr. Marshall H. Webb

**Published:** 1883-03

**Authors:** E. Osmond


### The Late Dr. Marshall H. Webb.

### A SUGGESTION.

From reading the eulogy on the late Dr. Marshall H. Webb in the Feburary No. of the *Dental Cosmos*, I am sorry to learn that he has left a widow and three children unprovided for, and that this is owing to the fact that he neglected his office practice, in order to teach others the art of filling teeth. As the late Dr.

Webb used the electric mallet exclusively, and spent his time in instructing others how to use it, and as all the patents on said electric mallet belong to the S. S. White Manufacturing Co., and they receive all the profits on the same; and furthermore, as they are about to publish a work by the late Dr. Webb, written on his death-bed, would it not be well for the said S. S. White Manufacturing Co. to give his widow and children all the profits from the sale of said work, and also a share of the electric mallet profits, instead of the *cheap* offer of Editor J. W. White, to become the treasurer of such sums as the profession may subscribe in their behalf? Empty eulogies will not fill the stomachs of the fatherless and the widow.

"Yes—such was the man, and so wretched his fate;
And thus, sooner or later, shall all have to grieve
Who waste their morn's dew in the beams of the Great,
And expect 'twill return to refresh them at eve.

"In the woods of the North, there are insects that prey
On the brain of the elk till his very last sigh.
Oh, Genius! thy patrons, more cruel than they,
First feed on thy brains, and then leave thee to die."

[MOORE.

Rich corporations are said to have no souls. Let us hope the S. S. White Manufacturing Co. will be found an exception.

E. OSMOND, M.D., D.D.S.

